# Chronic and intermittent administration of systemic nitroglycerin in the rat induces an increase in the gene expression of CGRP in central areas: potential contribution to pain processing

**DOI:** 10.1186/s10194-018-0879-6

**Published:** 2018-07-13

**Authors:** Rosaria Greco, Chiara Demartini, Anna Maria Zanaboni, Cristina Tassorelli

**Affiliations:** 1 Laboratory of Neurophysiology of Integrative Autonomic Systems, Headache Science Centre, IRCCS Mondino Foundation, Pavia, Italy; 2 Department of Brain and Behavioral Sciences, University of Pavia, Pavia, Italy

**Keywords:** Nitroglycerin, Topiramate, CGRP, Trigeminal nociception

## Abstract

**Background:**

Calcitonin gene related peptide (CGRP) is a key neuropeptide involved in the activation of the trigeminovascular system and it is likely related to migraine chronification. Here, we investigated the role of CGRP in an animal model that mimics the chronic migraine condition via repeated and intermittent nitroglycerin (NTG) administration. We also evaluated the modulatory effect of topiramate on this experimental paradigm. Male Sprague-Dawley rats were injected with NTG (5 mg/kg, i.p.) or vehicle, every 2 days over a 9-day period (5 total injections). A group of animals was injected with topiramate (30 mg/kg, i.p.) or saline every day for 9 days. Twenty-four hours after the last administration of NTG or vehicle, animals underwent tail flick test and orofacial Von Frey test. Rats were subsequently sacrificed to evaluate c-Fos and CGRP gene expression in medulla-pons region, cervical spinal cord and trigeminal ganglia.

**Results:**

NTG administration induced spinal hyperalgesia and orofacial allodynia, together with a significant increase in the expression of CGRP and c-Fos genes in trigeminal ganglia and central areas. Topiramate treatment prevented NTG-induced changes by reversing NTG-induced hyperalgesia and allodynia, and inhibiting CGRP and c-Fos gene expression in all areas evaluated.

**Conclusions:**

These findings point to the role of CGRP in the processes underlying migraine chronification and suggest a possible interaction with gamma-aminobutyrate (GABA) and glutamate transmission to induce/maintain central sensitization and to contribute to the dysregulation of descending pain system involved in chronic migraine.

## Background

Pain persistence is associated with peripheral and central nervous system reorganization involving neuronal and glial changes [1]. Migraine chronification may result from maladaptive neuroplasticity along the nociceptive pathway [2]. Repeated trigeminal activation at the meningeal neurovascular endings indeed, with the associated neurogenic inflammation, may induce peripheral and central sensitization, which in turn predisposes patients to develop more migraine attacks in a vicious cycle that, in some migraineurs, leads to chronic migraine [3]. The mechanisms involved in migraine chronification are largely elusive; however, a major role seems to be played by the neuropeptide calcitonin gene-related peptide (CGRP), a vasodilatory peptide released by trigemino-vascular endings to cause vasodilation, neurogenic inflammation and peripheral sensitization [4]. The role of CGRP in migraine attacks is reinforced by the efficacy of CGRP antagonism in animal models of migraine pain [5–7] and by promising reports on the efficacy of CGRP-related drugs in clinical trials, e.g. telcagepant or LY2951742 [8–10]. Recently, monoclonal antibodies directed against CGRP have proved effective in the preventive treatment of chronic migraine [11]. The precise mechanisms and sites where CGRP may act to favor chronification of migraine are still to be identified. CGRP receptors are largely distributed also in the brain, providing a wide range of possible CGRP targets and sites of interactions with other systems [12]. Topiramate is an antiepileptic drug with established efficacy in chronic migraine prevention [13, 14]. The drug likely acts on the cell excitatory mechanisms via its influence on the receptor/channel protein complexes [15–17].

Nitroglycerin (NTG) is a nitric oxide (NO) donor that has been used for years as a provocative test in migraine for diagnostic and research purposes [18, 19]. In rodents, NTG induces an increased sensitivity to pain stimuli [20–22] and its chronic and intermittent administration causes acute plantar mechanical hyperalgesia with a progressive and sustained hyperalgesia [23, 24]. This behavior nicely reflects the increased sensitivity to painful stimuli in migraineurs [25] and therefore may be relevant for understanding the mechanism underlying migraine. Here, we aimed at gathering more insights into the role and mechanisms of CGRP in migraine chronification by chronic migraine-like rat model that mimics the condition of chronic migraine. In this model we evaluated: a) CGRP and c-Fos gene expression in areas involved in trigeminal nociception; b) the nociceptive threshold at the tail flick; c) orofacial mechanical allodynia; d) the modulatory effect of the migraine preventive drug topiramate.

## Methods

### Animals

We used adult male Sprague-Dawley rats (weight 200-250 g, Charles River, s.r.l, Calco, Lecco, Italy) at the Centralized Animal Facility of the University of Pavia. The animals were housed under standard laboratory conditions in plastic boxes in groups of 2 with water and food available ad libitum and kept on a 12:12 h light-dark cycle, at room temperature of 19–21 °C with relative humidity of 70–80%. All procedures were conducted in accordance with the European Convention for Care and Use of Laboratory Animals and with the IASP’s guidelines for pain research in animals [26]. The experimental protocols were approved by the Italian Ministry of Health (Document number 1239/2015PR).

### Drugs

Nitroglycerin (NTG) (Bioindustria L.I.M. Novi Ligure (AL), Italy) was prepared from a stock solution of 5.0 mg/1.5 mL dissolved in 27% alcohol and 73% propylene glycol. For the injections, NTG was further diluted in saline (0.9% NaCl) to reach the final concentration of alcohol 6% and propylene glycol 16%. The diluted NTG is injected intraperitoneally (i.p.) at the dose of 5 mg/Kg [24, 27]. An equivalent volume of saline (0.9% NaCl), alcohol 6% and propylene glycol 16%) was used as vehicle. Topiramate (Topamax, Janssen-Cilag Cologno Monzese (MI), Italy) was dissolved in saline and administered i.p. at the dose of 30 mg/Kg [28]. Before baseline testing, rats were assigned to treatment groups according to a randomization list to ensure blinding to treatments of the researchers who performed the behavioral testing (tail flick test and Von Frey test, see below). Before testing the animals, the blinded examiners were instructed to observe the animal behavior, in order to evaluate the possible impact of topiramate on sedation or locomotor activities.

### Experimental design and experimental groups

**Fig. 1:**
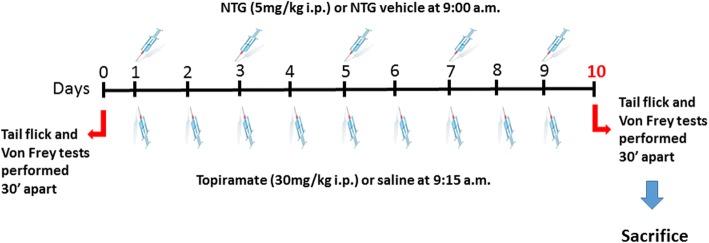
Experimental design and drugs administration schedule

An a priori power analysis was conducted to determine the minimal sample size needed to obtain a statistical power of 0.80 at an alpha level of 0.05. On the basis of our previous studies in rats evaluating the latency to the tail flick (sec.) before and after NTG, we calculated an effect size of 1.6 for this variable (GPower 3.1.9.2), estimating a sample size of at least 5 rats for experimental group. Rats were injected with NTG or vehicle, every 2 days over a 9-day total period (5 total injections). A group of animals was also injected with topiramate (TOP) or vehicle (saline) every day for 9 days. At baseline (day 0) and 24 h after the last administration of NTG (day 10), the animals belonging to the different experimental groups (see below) underwent the evaluation of the nociceptive threshold by means of the tail flick test and of the orofacial mechanical allodynia by means of the Von Frey test. The schedule of drug treatments is reported in Fig. 1.

*Experimental Groups***:** CT = NTG vehicle + TOP vehicle (*N* = 10–13 per group); NTG = NTG + TOP vehicle (*N* = 9–12 per group); TOP = NTG vehicle + Topiramate (*N* = 5–8 per group); NTG + TOP = NTG + Topiramate (N = 5–8 per group).

### Behavioral tests

Rats underwent tail flick test and Von Frey test 30 min apart from each other at baseline and on day 10.

#### Tail flick test

To determine thermal sensitivity, rats were gently restrained while an infrared light beam with a temperature of 50 °C was focused on the animal’s tail. The latency of retraction of the tail from the window of the light beam was automatically captured and measured (in seconds) by means of a sensor (Ugo Basile, model 7360, Varese, Italy) [20, 21]. A cut-off limit of exposure of 20 s was set to avoid tissue damage. The final latency value for each animal was calculated as the mean of four measurements in 4 different parts of the tail, specifically at 1, 3, 5 and 7 cm from the tip.

#### Von Frey test

For three consecutive days, prior to baseline testing, the rats were habituated to the behavioral test procedure. Rats were put in clear acrylic cages (30 × 30 × 20 cm) and the orofacial area of the animal was stimulated with a series of Von Frey filaments (bending force ranging from 0.02 to 6 g). Progressively increasing filament forces were applied (each of them 5 times every 30 s) to the cutaneous area on both the left and right sides of the face, over the rostral portion of the eye for periorbital testing and on the skin over the masseter muscle for jaw testing until we obtained a positive response, defined as one of the following: head withdrawal, face wipe, escape/attack. In the absence of a positive response to the starting filament (0.4 g), a heavier filament was applied whereas, with a positive response a lighter filament was tested. The mechanical threshold corresponded to the force of the filament that induced three positive responses [29, 30].

### CGRP and c-Fos gene expression

On day 10, immediately after undergoing the Von Frey test, all rats were sacrificed and their medulla-pons region, cervical spinal cord (C1-C2) and trigeminal ganglia were immediately chopped into parts for the evaluation of CGRP and c-Fos gene expression. mRNA levels were analyzed by real-time polymerase chain reaction (RT-PCR) as previously described [22, 31]. Primer sequences of Calca gene, coding for CGRP, (forward primer: CAGTCTCAGCTCCAAGTCATC; reverse primer: TTCCAAGGTTGACCTCAAAG) and c-Fos (forward primer: TACGCTCCAAGCGGAGAC; reverse primer: TTTCCTTCTCTTTCAGTAGATTGG) were obtained from the AutoPrime software (http://www.autoprime.de/AutoPrimeWeb). We used glyceraldehyde 3-phosphate dehydrogenase (GAPDH; forward primer: AACCTGCCAAGTATGATGAC; reverse primer: GGAGTTGCTGTTGAAGTCA) as housekeeping gene. All samples were assayed in triplicate. Gene expression was calculated using the ΔΔCt method.

### Data analysis and statistical evaluation

Our data showed a normal distribution when analyzed with the Kolmogorov–Smirnov (KS) normality tests. For the behavioral tests the inter-groups and within groups differences between baseline and post-treatment timings were analyzed using the Two-way ANOVA for repeated measures followed by Bonferroni post hoc test. For gene expression, the differences between groups were analyzed by One-way ANOVA followed by Newman–Keuls multiple comparison test. A *p* value < 0.05 was considered statistically significant. The results were reported in the figures and all data were expressed as the mean + standard error of the mean. The Statistical analysis was performed using GraphPad Prism 5.02.

## Results

### Behavioral testing

No distinctive pattern of behavior was identified in the different experimental groups by comparing the reports of the blinded observers after treatment unblinding.

#### Tail Flick test

**Fig. 2:**
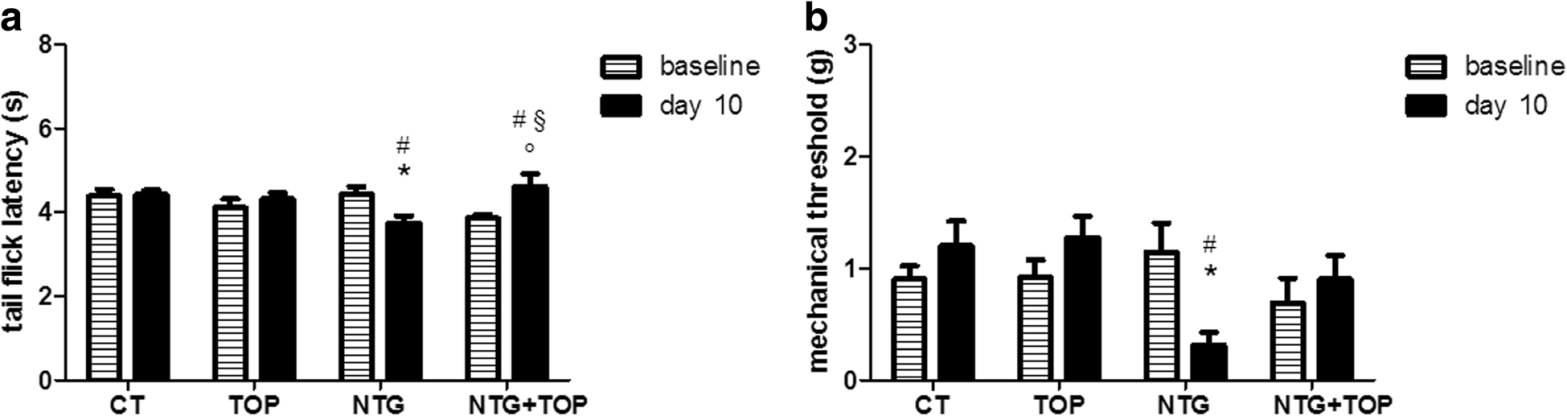
**a** Nociceptive thermal threshold (tail flick test) and **b** orofacial mechanical allodynia (Von Frey test) following chronic and intermittent NTG administration and chronic topiramate (TOP) treatment in rats. CT = NTG vehicle + TOP vehicle (*n* = 13); NTG = NTG + TOP vehicle (*n* = 12); TOP = NTG vehicle + Topiramate (*n* = 8); NTG + TOP = NTG + Topiramate (*n* = 8). Data are expressed as mean + SEM; two-way ANOVA followed by Bonferroni post hoc tests, F = 6.019 for tail flick test; F = 4.091 for Von Frey test. *p < 0.05 vs NTG baseline; °*p* < 0.05 vs NTG + TOP baseline; #*p* < 0.05 vs CT and TOP 1 day 10; §*p* < 0.05 vs NTG day 10

No significant difference among CT, NTG and NTG + TOP groups was observed at baseline (Fig. 2a). Chronic NTG administration caused a state of hyperalgesia, which was detected as a reduction in the latency at the tail flick test performed on day 10 as compared with both baseline value and CT group. The chronic administration of topiramate did not influence the tail flick latency, but it prevented NTG-induced hyperalgesic state. Data are reported in Fig. 2a.

#### Von Frey test

We did not detect any significant difference in baseline mechanical sensitivity among experimental groups (Fig. 2b). Chronic and intermittent NTG administration caused orofacial allodynia with a reduction in the mechanical pain threshold compared to the baseline value and to the CT group. Topiramate did not change the mechanical pain threshold when compared to the baseline value and to the CT group, but it prevented the development of NTG-induced allodynia. The mechanical threshold of NTG + TOP was increased compared to NTG group, although it did not reach a statistical significant level, probably because of the great variability of data. Data are reported in Fig. 2b.

### mRNA expression of CGRP and c-Fos genes

**Fig. 3:**
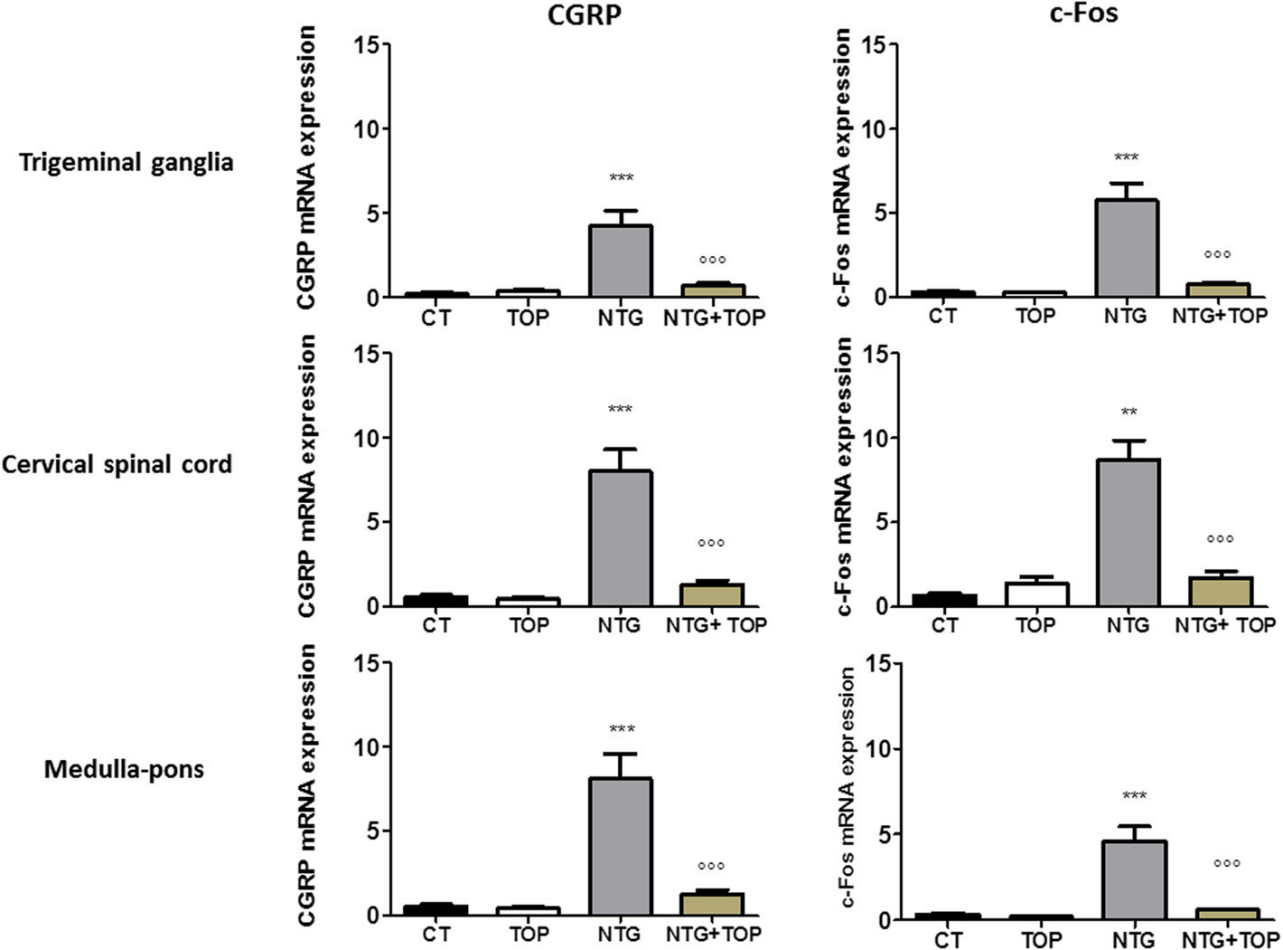
CGRP and c-Fos mRNA expression in areas involved in trigeminal nociception, following chronic and intermittent NTG administration in rat. CT = NTG vehicle + TOP vehicle (*n* = 10); NTG = NTG + TOP vehicle (*n* = 9); TOP = NTG vehicle + Topiramate (*n* = 5); NTG + TOP = NTG + Topiramate (*n* = 5). Data are expressed as mean + SEM. One way ANOVA followed by Newman-Keuls Multiple Comparison Test **p < 0.01 and ***p < 0.001 vs CT and TOP; °°°*p* < 0.001 vs NTG

NTG administration induced a significant increase in the expression of c-Fos and CGRP mRNA in the medulla-pons region, cervical spinal cord and trigeminal ganglia (Fig. 3). Topiramate did not modify genes expression in these areas when used alone, but it markedly attenuated CGRP and c-Fos gene expression induced by NTG in all the areas under evaluation (Fig. 3).

## Discussion

Migraine is a highly disabling condition [32] that manifests with recurring attacks. In 2–3% of the general population it becomes chronic (≥ 15 days/months) and therefore even more disabling. Progressive changes in nociceptive thresholds and subsequent central sensitization due to recurrent migraine headaches in vulnerable subjects contribute to the chronic migraine state. Understanding the mechanisms of chronification seems therefore extremely useful. The role of CGRP in the precipitation of migraine attacks is nowadays well accepted [33]. Less known is CGRP role in the processes that lead to migraine chronification.

Several studies indicate that NTG administration in rodents is a predictive model of migraine in humans [24, 34, 35]. In this study we aimed at developing and testing a chronic migraine-like model in rats, based on chronic and intermittent administration of NTG. In this model the animals become hyperalgesic and allodynic, which nicely mimics the clinical picture on one side [25, 36–39], and, on the other, parallels the findings obtained by Pradhan et al. in the animal model of chronic migraine devised in mice [24]. The availability of an animal model of chronic migraine in rats provides a precious opportunity for investigating the pathophysiological mechanisms involved in chronic migraine for a dual reason: 1) rat models have technical and practical advantages [40] and 2) the rat-based migraine model of acute NTG administration is well-established and has already yielded a wealth of data from several groups [41, 42].

Using this chronic migraine-like model we showed that the observed changes in pain perception – namely a reduction in the latency of the tail flick test and orofacial mechanical allodynia - are paralleled by an increased expression in CGRP mRNA in the trigeminal ganglia. CGRP is involved in the maintenance of ongoing sensitization and the increased gene expression observed in our model may be interpreted as a compensatory mechanism for reintegrating CGRP released at the trigeminovascular endings [43–45]. In agreement, Farajdokht et al. [46] demonstrated that the increase in CGRP gene expression in trigeminal ganglia is associated with increased transient receptor potential vanilloid type-1 mRNA levels in the same area 48 h after NTG injection. Previous reports have suggested that NTG induces hyperalgesia via complex mechanisms that involve also sensitization of the trigeminovascular system at the meningeal level [47–49]. Previously, we reported that acute NTG administration did not cause any change in CGRP-immunoreactivity in the lumbar dorsal horns, whereas it increased SP staining intensity in both the cervical and lumbar spinal cord 1 h after NTG administration [43]. Thus, though the effect of NTG administration seems relevant for processes that take place in the cervical spinal cord, we cannot rule out the occurrence of systemic NTG effects on other neuronal subpopulations and other areas of the nervous system. Of note, in a previous study Edelmeyer et al. [50] showed that inflammatory mediators applied to the dura of non-anesthetized rats caused strong and time-related facial and hindpaw allodynia, thus suggesting that the alterations in pain sensitivity associated with primary headache, probably reflect a more generalized condition of central sensitization [50].

In a previous study, we showed that acute NTG administration induces an increase in CGRP gene expression in the trigeminal ganglia, cervical spinal cord and medulla-pons area 5 h after the administration [31, 51]. Here, we show that the levels of CGRP mRNA are elevated in the same areas 24 h after the last dose of NTG administration. These findings are consistent with the synthesis and release of CGRP in these central areas, where the peptide can modulate nociceptive transmission [52]. Indeed, CGRP mRNA signal has been detected in the nucleus trigeminal caudalis (NTC) and other brain structures of naïve rats [53, 54], which include the nucleus tractus solitarius, receiving inhibitory baroreceptor afferents, the ventrolateral medulla, a key structure in the descending pain modulation, and the pontine parabrachial nucleus (PBN) that contributes to nociceptive transmission with its projections to the central nucleus of the amygdala (CeA) [55]. More importantly, CGRP and its receptor antagonists act on neurons in the ventrolateral periaqueductal gray to influence nociceptive transmission in the nucleus tractus caudalis. Functional CGRP receptors are present as well in other regions of the brain that are involved in the modulation of migraine pain [56]. Taken together, these observations support the contribution of CGRP in central sensitization and in the functioning of descending pain pathways, both of which are implicated in chronic migraine [57].

To further test the relevance of our animal model of chronic migraine we evaluated the effect of chronic administration of topiramate, one of the few drugs that have proved effective in the preventive treatment of chronic migraine in randomized controlled trials [58, 59].

Topiramate reduces brain hyperexcitability, a hallmark of migraine [60], via multiple mechanisms: blockade of voltage dependent sodium channels, increased activity of the neurotransmitter gamma-aminobutyrate (GABA) and antagonism of the α-amino-3-hydroxy-5-methyl-4-isoxazolepropionic acid (AMPA)/kainate subtype of the glutamate receptor and inhibition of the carbonic anhydrase enzyme [61, 62]. An in vitro study demonstrated that topiramate is able to attenuate neurogenic dural vasodilation by inhibiting the release of CGRP from prejunctional trigeminal neurons, thus suggesting an interaction with this neuropeptide [15].

Chronic treatment with topiramate in our model reduced trigeminal spinal hyperalgesia and mechanical allodynia, while diminishing CGRP mRNA levels in the areas under investigation. This finding seems in agreement with preclinical studies showing that topiramate has an anti-allodynic effect in neuropathic pain at doses of 20–50 mg/kg [28, 63–65]. Chronic administration of topiramate (50 mg/kg/day, i.p.) to neuropathic rats diminished the mechanical sensitivity and shortened the period of allodynia to 8 days [28].

CGRP can directly activate the nociceptors or facilitate their firing, thus causing peripheral sensitization and hyperalgesia [1]. In this frame, it is possible that the anti-hyperalgesic effect of topiramate observed in the present study is related to the inhibitory activity on the neuronal firing within the trigeminocervical complex and the ventroposteromedial thalamic nucleus, as reported by Andreou and Goadsby [66].

Topiaramate blocks voltage-dependent sodium channel in spinal cord neurons, which prompts a possible mechanism for reducing afferent input to the NTC [67]. Other mechanisms could be involved such as anti-inflammatory and immunomodulatory effects or inhibition of neurotransmitters release [68, 69]. Central sensitization involves an increased sensitivity of second-order neurons to afferent inputs, the augmentation of receptive fields and an increased excitability; furthermore, it involves long-term effects related to transcriptional changes and dysfunctions in the descending pain system [70]. The maintenance of central sensitization may be related to the activation of the glutamate N-methyl-D-aspartate (NMDA), AMPA and metabotropic receptors. Thus, AMPA/kainate receptors inhibition by topiramate treatment could decrease neuronal hyperexcitability within the NTC and brain structures receiving inputs from second order neurons [68]. Glutamate-like immunoreactivity has been indeed detected in tooth pulp neurons that project to the NTC in the rat [71].

It is worth noting that in our experimental paradigm, topiramate treatment significantly reduced CGRP gene expression in cervical spinal cord and medulla-pons. In agreement, topiramate treatment repressed KCl stimulated CGRP release in a time and concentration dependent manner in trigeminal cultures [72]. However, since GABAergic mechanisms have been shown to be implicated in the trigeminocervical complex, it is stimulating to hypothesize that GABA potentiation due to topiramate treatment may interfere with CGRP synthesis in the NTC, as well as in the areas involved in the descending pain system [34, 35, 57, 73]. Synaptic contacts between CGRP-positive terminals and GABAergic neurons were found within the CeA, which may underlie the pain-related neural pathway from PBN to CeA, in chronic pain modulation.

The increased c-Fos mRNA levels found 24 h after NTG treatment, confirms that NTG-induced transcriptional changes are not limited to peripheral areas, but rather affect central structures, directly or indirectly, thus reflecting a widespread, peripheral and central diffusion of sensitization phenomena consistent with the observed alterations in pain responses. While a large body of information has been collected over the years by our group and others on the acute NTG model, the mechanisms responsible for c-Fos gene expression after chronic NTG are almost completely unknown. At this time, we can only speculate that the model proposed in this study actually engages the CNS with persisting functional and morphological changes associated to chronic sensitization.

### Limitations of the study

The design of study foresaw the chronic administration of topiramate. Data from the literature suggest that acute administration of topiramate reduces neuronal firing in the trigeminovascular complex [66] therefore the chronic administration used in our design might not have been necessary. In the absence of reliable evidence, we selected the chronic administration for several reasons. First, this treatment schedule reflects more closely the clinical scenario, where topiramate is used daily over months to prevent migraine attacks. Second, data is lacking on the serum/plasma concentration of topiramate in the rat. Human studies show that the plasma half-life of the drug is 20–30 h after a single oral administration [74, 75], while it is shorter in rats [76], thus posing the issue of unstable blood levels of topiramate. This could have been addressed by increasing the dose of the single administration, but also the possibility that the sedative effect of such a higher dose might interfere with the nocifensive behavior of the rat [77]. The lack of precise pharmacokinetics studies on repeated dosing in the laboratory animal ultimately suggested the selection of dose regimens based on a best-guess modality, guided by careful evaluation of available evidence.

Future studies are needed to address topiramate serum/plasma concentrations in our model, together with the evaluation of the potential impact of topiramate-induced locomotor activity in the nocifensive behavior of chronically NTG-treated rats using specific behavioral testing.

## Conclusions

The present findings, obtained in a novel chronic migraine-like model in the rat, suggest that CGRP pathway activation is involved in the facilitation of nociceptive transmission and is likely to contribute to central sensitization and dysfunction in descending pain control, via modulatory effects that encompass the modulation of voltage dependent sodium channels, GABA activity and the glutamate pathway. A fuller understanding of CGRP effects and CGRP network in brain regions will provide powerful insights for understanding the complex circuitry of migraine and for the improvement of migraine therapies.

## Abbreviations

AMPA: *α*-amino-3-hydroxy-5-methyl-4-isoxazolepropionic acid
CeA: Central nucleus of the amygdala
CGRP: Calcitonin gene-related peptide
GABA: Gamma-aminobutyrate
GAPDH: Glyceraldehyde 3-phosphate dehydrogenase
NMDA: N-methyl-D-aspartate
NO: Nitric oxide
NTC: trigeminal nucleus caudalis
NTG: Nitroglycerin
PBN: Pontine parabrachial nucleus
RT-PCR: Real-time polymerase chain reaction
TOP: Topiramate
